# Bnip3 Binds and Activates p300: Possible Role in Cardiac Transcription and Myocyte Morphology

**DOI:** 10.1371/journal.pone.0136847

**Published:** 2015-08-28

**Authors:** John W. Thompson, Jianqin Wei, Kweku Appau, Huilan Wang, Hong Yu, Maria G. Spiga, Regina M. Graham, Keith A. Webster

**Affiliations:** 1 Department of Molecular and Cellular Pharmacology and the Vascular Biology Institute, University of Miami Miller School of Medicine, Miami, Florida, United States of America; 2 Department of Cardiology, Second Affiliated Hospital, College of Medicine, Zhejiang University, Hangzhou, China; University of Cincinnati, College of Medicine, UNITED STATES

## Abstract

Bnip3 is a hypoxia-regulated member of the Bcl-2 family of proteins that is implicated in apoptosis, programmed necrosis, autophagy and mitophagy. Mitochondria are thought to be the primary targets of Bnip3 although its activities may extend to the ER, cytoplasm, and nucleus. Bnip3 is induced in the heart by ischemia and pressure-overload, and may contribute to cardiomyopathy and heart failure. Only mitochondrial-dependent programmed death actions have been described for Bnip3 in the heart. Here we describe a novel activity of Bnip3 in cultured cardiac myocytes and transgenic mice overexpressing Bnip3 in the heart (Bnip3-TG). In cultured myocytes Bnip3 bound and activated the acetyltransferase p300, increased acetylation of histones and the transcription factor GATA4, and conferred p300 and GATA4-sensitive cellular morphological changes. In intact Bnip3-TG hearts Bnip3 also bound p300 and GATA4 and conferred enhanced GATA4 acetylation. Bnip3-TG mice underwent age-dependent ventricular dilation and heart failure that was partially prevented by p300 inhibition with curcumin. The results suggest that Bnip3 regulates cardiac gene expression and perhaps myocyte morphology by activating nuclear p300 acetyltransferase activity and hyperacetylating histones and p300-selective transcription factors.

## Introduction

Bnip3 (Bcl-2/adenovirus E1B 19-kDa interacting protein 3) is a member of the BH3-only family of Bcl-2 proteins and has been assigned roles in apoptosis, programmed necrosis, autophagy and mitophagy during exposure of cells and tissues to hypoxia or ischemia (reviewed in [1–3]). Most studies report that the transmembrane (TM) domain is required for Bnip3-mediated cell death and the death signal can be initiated by directing Bnip3 to mitochondrial and non-mitochondrial sites [4, 5].

Pro-survival properties of Bnip3 that are unrelated to its regulation of mitochondrial functions have also been reported. Bnip3 was shown to regulate the activity of the mammalian target of rapamycin (mTOR1) by selectively binding the regulatory GTPase protein Rheb (Ras homolog enriched in brain) in cells exposed to hypoxia [6]. Bnip3 was also shown to confer survival signals in glioblastoma tumor cells by suppressing transcription of the apoptosis-inducing factor and death receptor 5 genes [7, 8].

In the heart Bnip3 has been assigned both death [9, 10] and survival promoting activities [11, 12]. Our group reported that Bnip3-mediated death is caspase-independent and requires concurrent hypoxia with acidosis [10]. Multiple studies have demonstrated elevated autophagy in cardiac myocytes and intact hearts during hypoxia, ischemia, ischemia-reperfusion, and heart failure including roles for Bnip3 [13, 14]. Overexpression of Bnip3 in transgenic mouse hearts confers increased apoptosis, contractile dysfunction and age-related dilated cardiomyopathy that culminate in heart failure [15]. Bnip3 expression is also induced in the heart by pressure overload through a c-Jun-N-terminal kinase (JNK)-FOXO3a pathway and contributes to enhanced cell death and cardiomyopathy by disrupting ER and mitochondrial calcium handling [16]. Therefore Bnip3 can promote cell death in the heart by targeting both mitochondria and ER. Here we provide evidence for another activity of Bnip3 in cardiac myocytes that is independent of mitochondria, ER or cell death, and involves targeting of histone acetyltransferase p300 and possibly cardiac-specific gene expression.

## Materials and Methods

### Ethics statement

All animal protocols were approved by the Animal Care and Use Committee of the University of Miami (assurance number: A-3224-01). All experiments were conducted in accordance to ARRIVE guidelines.

### Cell culture

Primary cultures of neonatal rat cardiac myocytes were prepared and exposed to hypoxia or hypoxia-acidosis as previously described [10] [17].

### Transgenic mice

Transgenic over-expression of Bnip3 in C57BL/6 mice was achieved by standard transgenic procedures using the α-MHC promoter to direct expression selectively to the myocardium. Three founders were backcrossed to wild type C57BL/6 mice for 3 generations to obtain stable lines. Cardiac functions were monitored using a Visual Sonics Vevo-770 imaging system (Toronto, Canada). Curcumin (100 mg/kg) or vehicle (1% gum arabic) was administered to animals once a day by gavage. Hearts were sectioned and stained as previously described [18].

### Western blot

Our western blot procedures are described in detail elsewhere [10, 19]. Antibodies include acetylated histone H3, acetylated lysine, p300, and GATA4 (EMD Millipore); acetylated histone H4 and Rheb (Cell Signaling); Bnip3 (Abcam), and MEF2 (Santa Cruz Biotechnology). Proteins were immunoprecipitated as previously described [18]. Western blots were quantified using Image J software.

### DNA fragmentation

Described in detail elsewhere [17]. Briefly, cells were lysed in a buffer containing 100 mM NaCl, 10 mM Tris-Cl (pH 8.0), 5 mM EDTA, 0.5% SDS, and 1 μg/mL proteinase K; proteins were precipitated with 0.8 M NaCl at 4°C overnight; and DNA extracted with phenol/chloroform and isopropanol. The pellet was resuspended in Tris-EDTA buffer. DNA samples (5 μg) were subjected to electrophoresis in 2% agarose gels and were imaged by ethidium bromide staining and digital photography.

### HAT and HDAC

HAT and HDAC activity were determined in whole cell lysates, in nuclear lysates incubated with recombinant Bnip3 protein or from p300 immunoprecipitate [18]. Briefly, hearts or cells were washed using ice cold PBS, minced and homogenized in lysis buffer. Cleared lysates were analyzed for HDAC and total or p300-specific HAT activity with commercially available kits (BioVision).

### Immunocytochemistry

Procedures are described in detail elsewhere [18, 20]. Briefly fixed cells on coverslips were exposed to antibodies against acetylated histone H3 or acetylated histone H4 overnight at 4°C followed by incubation with fluorescent conjugated secondary antibodies. TUNEL assays were performed using an *in situ* cell death detection kit (Roche Applied Science).

### Morphology

The size and shape of isolated neonatal rat cardiac myocytes was quantified as described previously [18]. Briefly, myocytes were plated on Nunc 2-well glass chamber slides and transfected 2 days later with the indicated plasmids. After 48 h the cells were fixed and myocyte morphology determined by immunocytochemistry using antibodies against T7 and troponin I to identify transfected cardiac myocytes. The primary major and minor axes of transfected myocytes were measured using Image J software. To quantify the effect of Rheb on myocyte size, freshly isolated myocytes were transfected with Rheb or random sequence (RS) siRNA and GFP and exposed to 20% fetal calf serum for 24 h to induce hypertrophy. Size was quantified using Image J by imaging GFP or phalloidin stain.

### Statistical analysis

All data is expressed as mean ± SEM of at least three experiments. Statistical analysis was performed using Social Sciences Statistical Package (SPSS, Inc.). Student t-test analysis was used for two-group comparison whereas multigroup analysis was performed using a one-way ANOVA analysis with Bonferroni’s correction. *p* < 0.05 was considered to be statistically significant.

## Results

### Hypoxia-acidosis confers histone hyperacetylation

**Fig 1:**
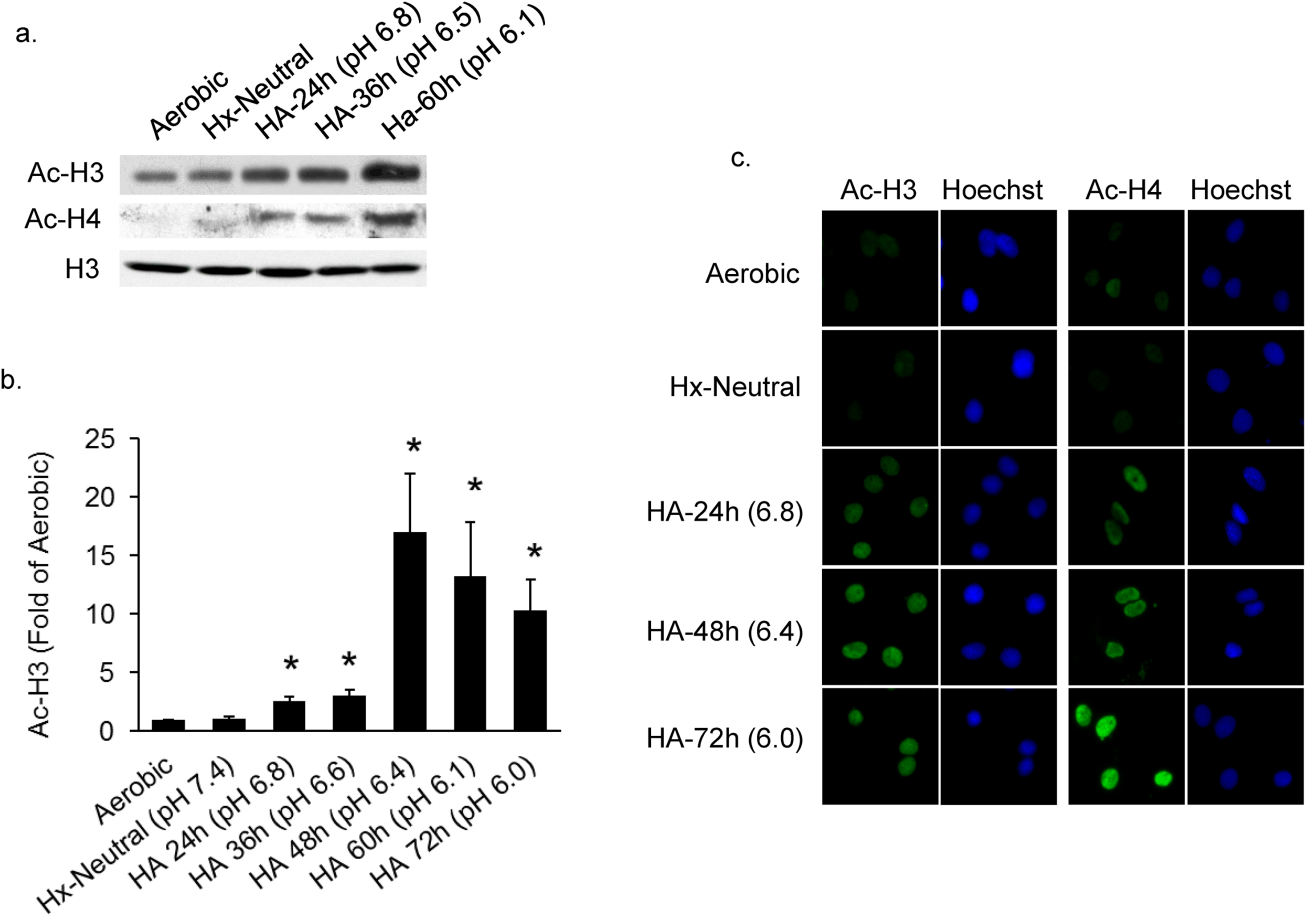
Hypoxia-acidosis increases histone acetylation. Cardiac myocytes were exposed to aerobic, hypoxic or hypoxia-acidosis (HA) conditions for the indicated times. In (A and B) histone H3 and H4 acetylation was quantified as described in Methods (n = 4; * p < 0.05). In (C) histone acetylation was imaged by immunocytochemistry of cardiac myocytes cultured as indicated. All results are mean ± S.E.M.

Simulation of ischemia by exposure of isolated cardiac myocytes to concurrent hypoxia with acidosis (HA) revealed a markedly increased acetylation of histones H3 and H4 compared with myocytes exposed to aerobic incubation (Fig 1A–1C). A maximal increase in H3 acetylation of >15-fold was observed at 48 h of hypoxia at a pH of 6.4 (Fig 1B). Fluorescence immunostaining of acetylated H3 and H4 confirmed these results showing a gradual increase of fluorescence over time in hypoxia and decreasing pH (Fig 1C).

### Histone hyperacetylation is independent of cell death

**Fig 2:**
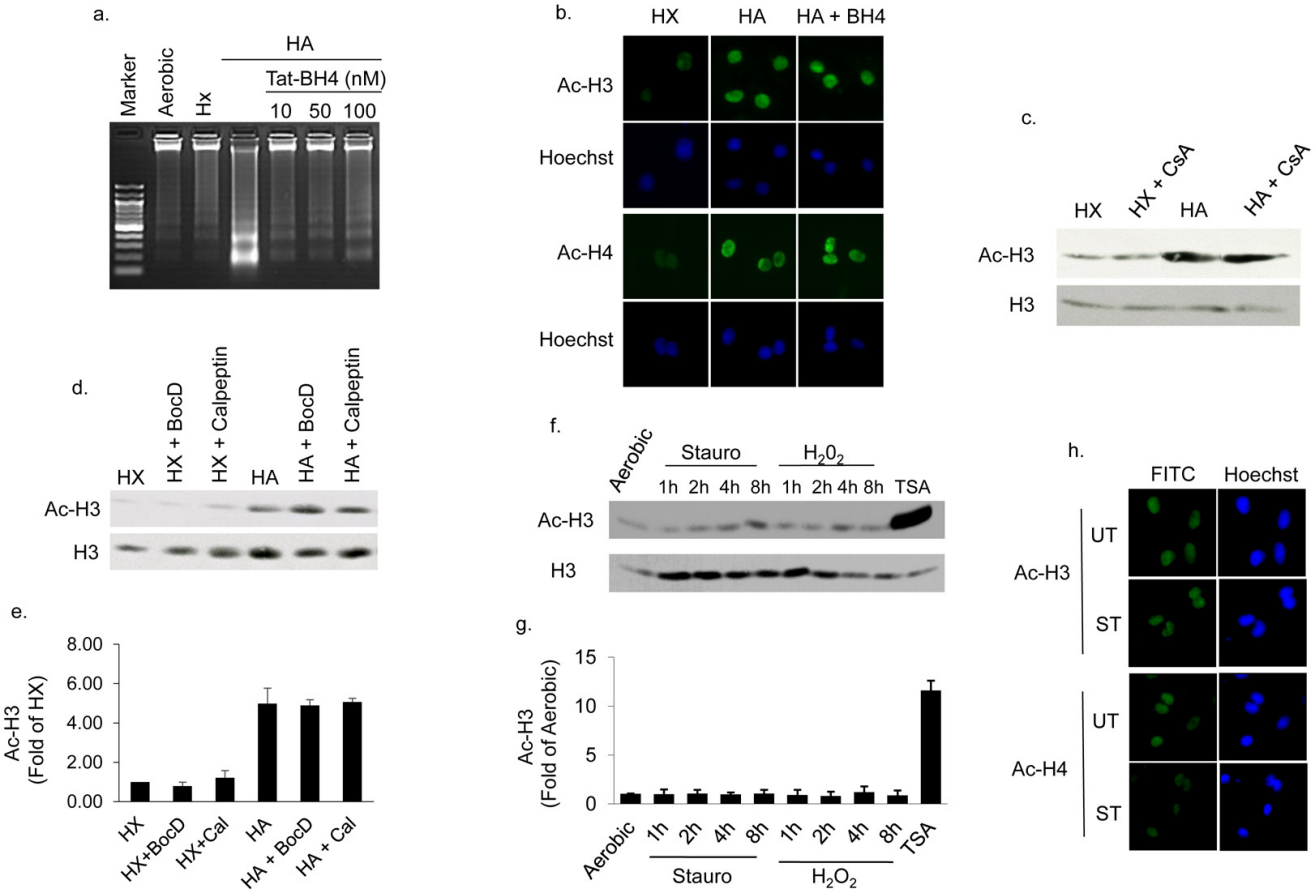
Histone hyperacetylation is independent of cell death. In (A and B), cardiac myocytes were pretreated with mitochondrial permeability transition pore (mPTP) inhibitor TAT-BH4, before exposure to hypoxia or HA, and histone acetylation or DNA fragmentation measured as described in Methods. In (C and D), cardiac myocytes exposed to hypoxia (HX) or hypoxia-acidosis (HA) in the presence and absence of the mPTP inhibitor cyclosporine A (CsA), the caspase inhibitor BocD, or the calpain inhibitor calpeptin as indicated. Western blot quantitation of histone H3 acetylation in the presence of BocD and calpeptin (Cal) is shown in (E). In (F and G) cardiac myocytes were treated with vehicle (UT), or with staurosporine or hydrogen peroxide (H_2_O_2_) as indicated, to induce cell death, and the acetylation level of histone H3 was determined by western blot analysis. As a positive control for histone acetylation cardiac myocytes were also treated with the HDAC inhibitor TSA. The effect of staurosporine (ST) treatment on histone H3 and H4 acetylation is shown in (H). All results are mean ± S.E.M., n = 3.

We previously reported that the death pathway initiated by HA requires Bnip3 activation [10] and proceeds in a caspase independent, calpain dependent and mPTP sensitive pathway. To determine whether histone hyperacetylation was a secondary consequence of cell death, cardiac myocytes were subjected to HA in the presence of caspase, calpain or mPTP inhibitors. As shown in Fig 2A and 2B), inhibition of the mitochondrial permeability transition pore (mPTP) with a TAT-BH4 peptide [21–23] significantly reduced DNA fragmentation, an indicator of apoptotic cell death (Fig 2A), without affecting histone hyperacetylation (Fig 2B). Similarly treating myocytes with the mPTP inhibitor cyclosporine-A, pan-caspase inhibitor BocD or calpain inhibitor calpeptin were unable to block histone H3 hyperacetylation during hypoxia-acidosis (Fig 2C–2E). Because both calpain and mPTP inhibitors block HA induced cell death, these result suggest that histone hyperacetylation occurs independently of cell death. To confirm that cell death alone was not responsible for enhancing histone hyperacetylation, we repeated the measurements of histone acetylation in cardiac myocytes treated with staurosporine or H_2_O_2_, agents that activate apoptotic and necrotic death pathways respectively. As shown in Fig 2F–2H), neither staurosporine nor H_2_O_2_ enhanced histone acetylation. In results not shown we also found that histone hyperacetylation was not observed in aerobic cultures made acidic by the addition of lactic acid. Therefore HA-mediated histone hyperacetylation is not a function of cell death, or exposure to hypoxic or acidotic conditions alone but is conferred preferentially by the HA combination.

### Histone hyperacetylation requires Bnip3

**Fig 3:**
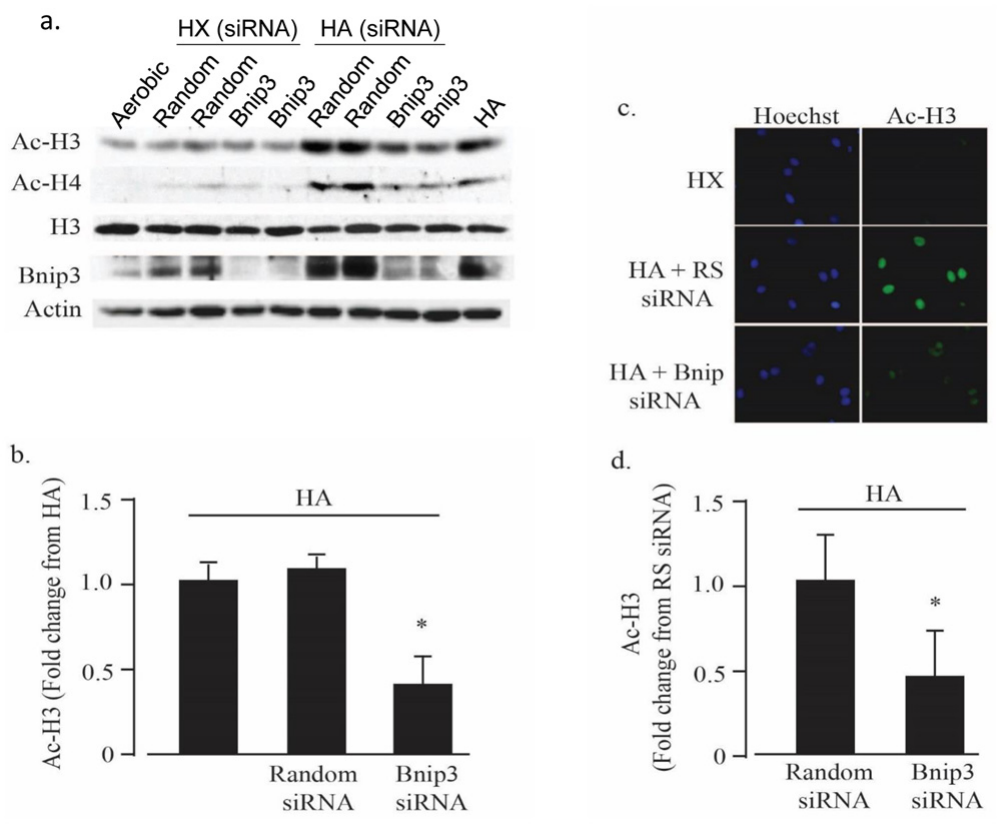
Bnip3 regulates histone acetylation during hypoxia-acidosis. In (A and B), cardiac myocytes were treated with random sequence or Bnip3 specific siRNA as indicated and histone acetylation determined by western blot analysis as described in Methods (n = 4; * p < 0.05). In (C and D), cardiac myocytes were subjected to hypoxia (HX) or hypoxia-acidosis (HA) in the presence of Bnip3 or random (RS) siRNA treatments as in (A and B) and the level of acetylated histone H3 determined by immunocytochemistry. Nuclei were labeled with Hoechst. (n = 4; * p<0.05). All results are mean ± S.E.M.

Bnip3 is induced by hypoxia and super-induced and activated by HA [10]. To investigate a possible role for Bnip3 in histone hyperacetylation we used Bnip3 selective siRNAs to knock down Bnip3 expression before subjecting cells to HA. As shown in Fig 3A and 3B, Bnip3 siRNA reduced Bnip3 expression >70% and concurrently blocked histone acetylation levels by a similar extent. These results were confirmed by immunocytochemistry analysis in Fig 3C and 3D that demonstrate a significant reduction in histone H3 acetylation during HA in the presence of Bnip3 siRNA. Taken together, the results described in Figs 1–3 indicate that HA-mediated histone hyperacetylation 1) requires Bnip3 and 2) occurs in parallel but independently of cell death during exposure of cardiac myocytes to HA.

### Histone acetylation during HA exposure involves p300-HAT

**Fig 4:**
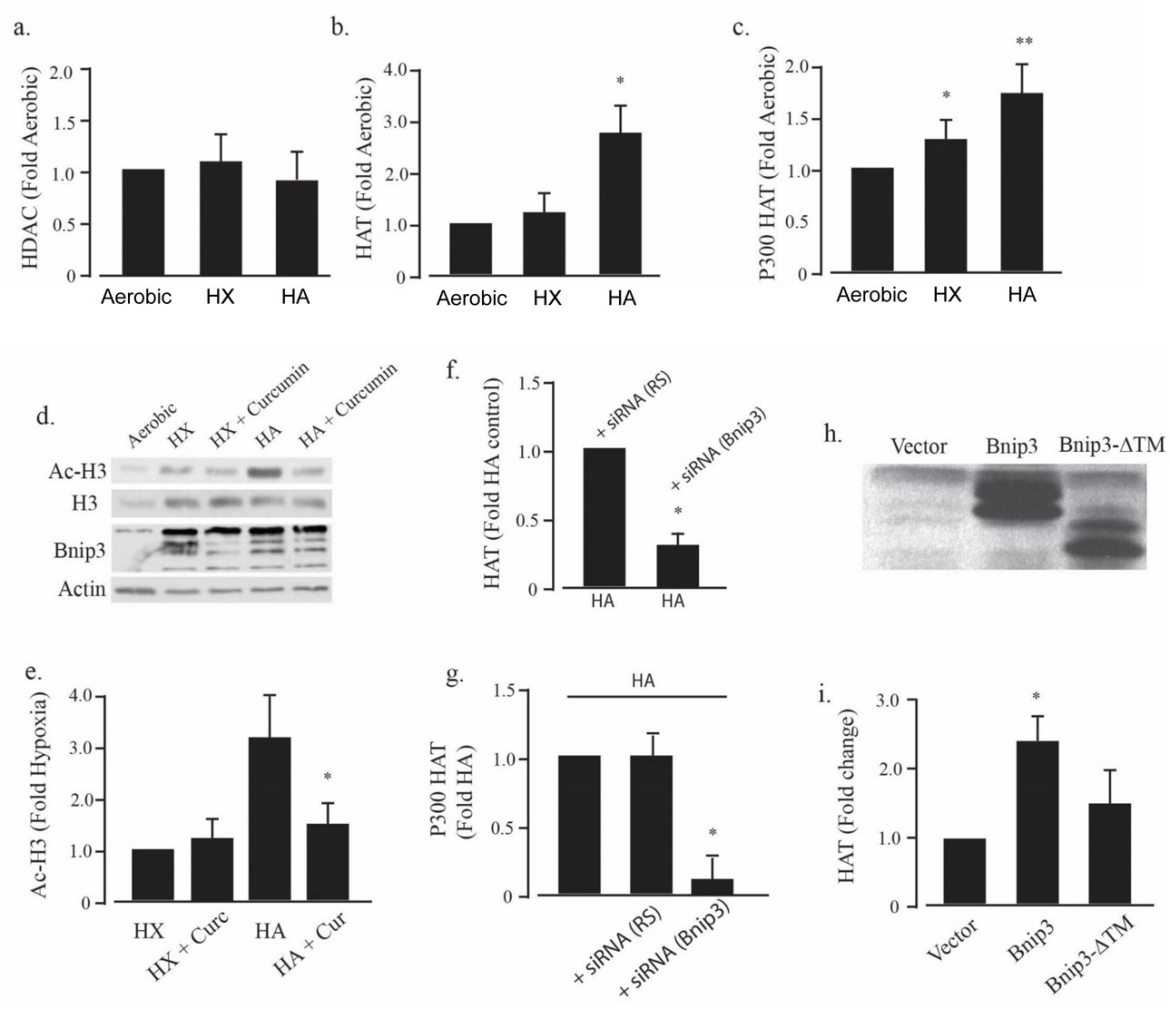
Bnip3 increases p300 acetyltransferase activity. Histone deacetylase (HDAC) (A) and acetyltransferase (HAT) (B) activities were measured in cardiac myocytes exposed to aerobic, hypoxia (HX) and hypoxia-acidosis (HA) as described in Methods. P300-specific acetyltransferase activity is shown in (C). The effect of the p300 inhibitor, curcumin, on histone H3 acetylation (Ac-H3), and Bnip3 protein levels during hypoxia and HA is shown in (D). Western blot quantitation of histone H3 acetylation levels in the presence of curcumin (Cur) is shown in (E). In (F and G), cardiac myocytes were treated with random (RS) or Bnip3 specific siRNA and total cellular HAT (F) or p300 specific HAT (G) activities were determined during hypoxia-acidosis (HA). In (H and I) recombinant Bnip3 or transmembrane deletion mutant Bnip3 (Bnip3-ΔTM) protein were incubated with nuclear lysates from normoxic cardiac myocytes as described in Methods and the HAT activity determined. Results are mean ± S.E.M., * significantly different then controls (p < 0.05), n = 3.

We next investigated the molecular mechanism by which Bnip3 regulates histone acetylation. The balance of activities of histone acetyltransferase (HAT) and histone deacetylase (HDAC) enzymes determines the level of histone acetylation. Therefore we measured total cellular HAT and HDAC activities following exposure to aerobic, hypoxic or HA conditions. As demonstrated in Fig 4A and 4B, HDAC activity was unaffected by hypoxia or HA when compared to parallel cultures maintained in aerobic conditions. In contrast we observed a significant increase in HAT activity in cultures exposed to HA but not hypoxia-alone. Because increased p300 acetyltransferase activity is known to parallel cardiac growth and myocardial remodeling after ischemia, we hypothesized that p300-specific acetyltransferase activity may be increased during HA. To test this, p300 was immunoprecipitated from cardiac myocytes after exposure to aerobic, hypoxic or HA conditions and the activity quantified. As shown in Fig 4C there was a small but significant increase in p300-dependent acetyltransferase activity in myocytes exposed to hypoxia alone that was significantly augmented by HA. To determine whether increased p300 acetyltransferase activity was responsible for histone hyperacetylation during HA, cultures were pretreated with curcumin, a partially selective inhibitor of p300- acetyltransferase activity [24, 25]. As shown in Fig 4D and 4E curcumin treatment significantly blocked histone H3 hyperacetylation during exposure to HA without effecting Bnip3 expression. These results suggest that histone hyperacetylation during HA requires both p300 and Bnip3. To further investigate the role of Bnip3 in the regulation of HAT activity, myocytes were pretreated with a Bnip3-selective siRNA or random sequence siRNA, and the HAT activity determined in cultures exposed to HA. As shown in Fig 4F and 4G total HAT and p300-specific acetyltransferase activity were both significantly reduced by Bnip3 knockdown when compared to random sequence siRNA treatments. These results confirm that Bnip3 is required for p300-activation by HA. To further characterize the mechanism of Bnip3-regulation of HAT activity, recombinant Bnip3 was incubated with nuclear lysates isolated from normoxic cardiac myocytes. As shown in Fig 4H and 4I, recombinant Bnip3 alone, in the absence of HA increased nuclear HAT activity. In contrast, a transmembrane deletion (ΔTM) mutant of Bnip3 was not effective, a result that suggests a requirement for the Bnip3 transmembrane domain.

### Bnip3 regulates acetylation of GATA4

**Fig 5:**
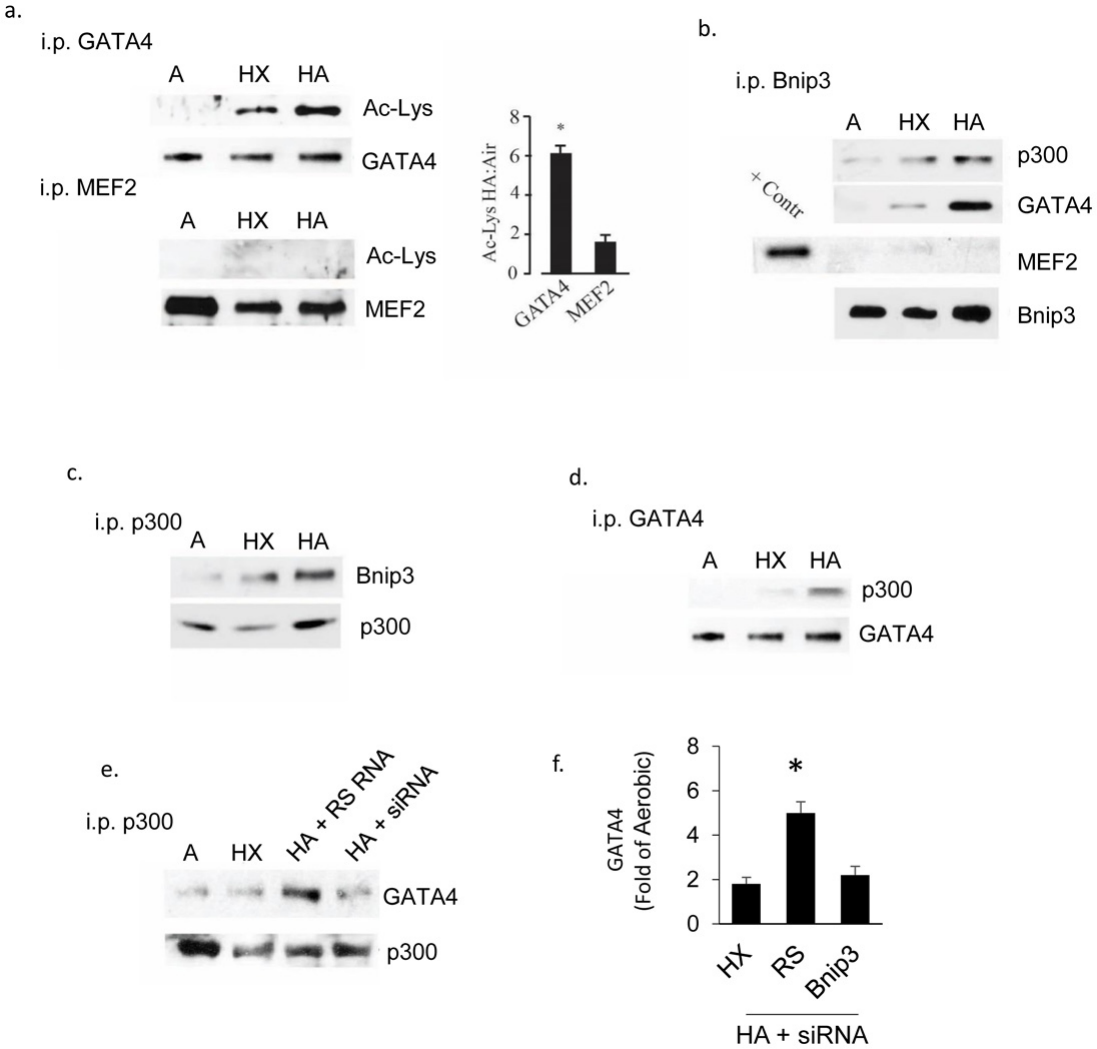
Bnip3 binds GATA4 and p300. GATA4 and MEF2 were immunoprecipitated from cardiac myocytes exposed to aerobic (A), hypoxia (HX) or hypoxia-acidosis (HA) as indicated and the acetylation level of each protein determined by western blot analysis (A). Western blot quantitation of changes in GATA4 and MEF2 acetylation levels in hypoxic-acidotic cardiac myocytes is shown to the right. In (B-D), Bnip3, GATA4 and p300 were immunoprecipitated from cardiac myocytes exposed aerobic (A), hypoxia (HX), or hypoxia-acidosis (HA). Precipitated proteins were detected by western blot analysis using antibodies directed against the indicated proteins. In (E) p300 was immunoprecipitated from cardiac myocytes treated with either random sequence siRNA (RS RNA) or Bnip3 specific siRNA (siRNA) and the western blot probed for GATA4. Western blot quantitation of immunoprecipitated GATA4 is shown to the right (F). Results are mean ± S.E.M., * significantly different then controls (p < 0.05), n = 3.

Acetyltransferases regulate gene expression by acetylating and modulating the activity of transcriptional regulators as well as histones. Our group as well as others has shown that activated p300 acetylates cardiac-specific factors involved in cardiac myocyte growth including the transcription factors GATA4 and MEF2 [18, 26–28]. To determine whether these transcription factors are targets for the enhanced p300 activity associated with elevated Bnip3 expression, we examined the acetylation levels of GATA4 and MEF2 from cardiac myocytes subjected to aerobic, hypoxia or HA. As shown in Fig 5A, acetylated GATA4 was increased in extracts from hypoxic cultures and this was enhanced by HA. In contrast, MEF2 acetylation was barely detectable under any condition, consistent with our previous findings in wild type mouse hearts [18]. To investigate possible protein-protein interactions we implemented reciprocal co-immunoprecipitations of Bnip3, p300 and GATA4 in cardiac myocytes subjected to aerobic, hypoxic or HA conditions. The results shown in Fig 5B–5D) are consistent with Bnip3-p300 and Bnip3-GATA4 interactions but negligible interaction of MEF2 with Bnip3. To determine whether Bnip3 enhanced the association of p300 with GATA4, cardiac myocytes were subjected to HA in the presence of Bnip3-siRNA or random sequence siRNA and the association of p300 and GATA4 determined again by p300 immunoprecipitation. As shown in Fig 5E, Bnip3-siRNA significantly reduced the pull-down of GATA4 by p300 in myocytes subjected to HA compared with random sequence RNA treated parallel cultures. These results indicate a possible physical interaction of Bnip3, p300 and GATA4. Whereas we were unable to detect any MEF2-Bnip3 interaction, it is possible that MEF2 forms weaker interactions with P300 and Bnip3 that are not realized by co-immunoprecipitation therefore such interactions cannot be ruled out.

### Curcumin and GATA4 sensitive changes in myocyte morphology

**Fig 6:**
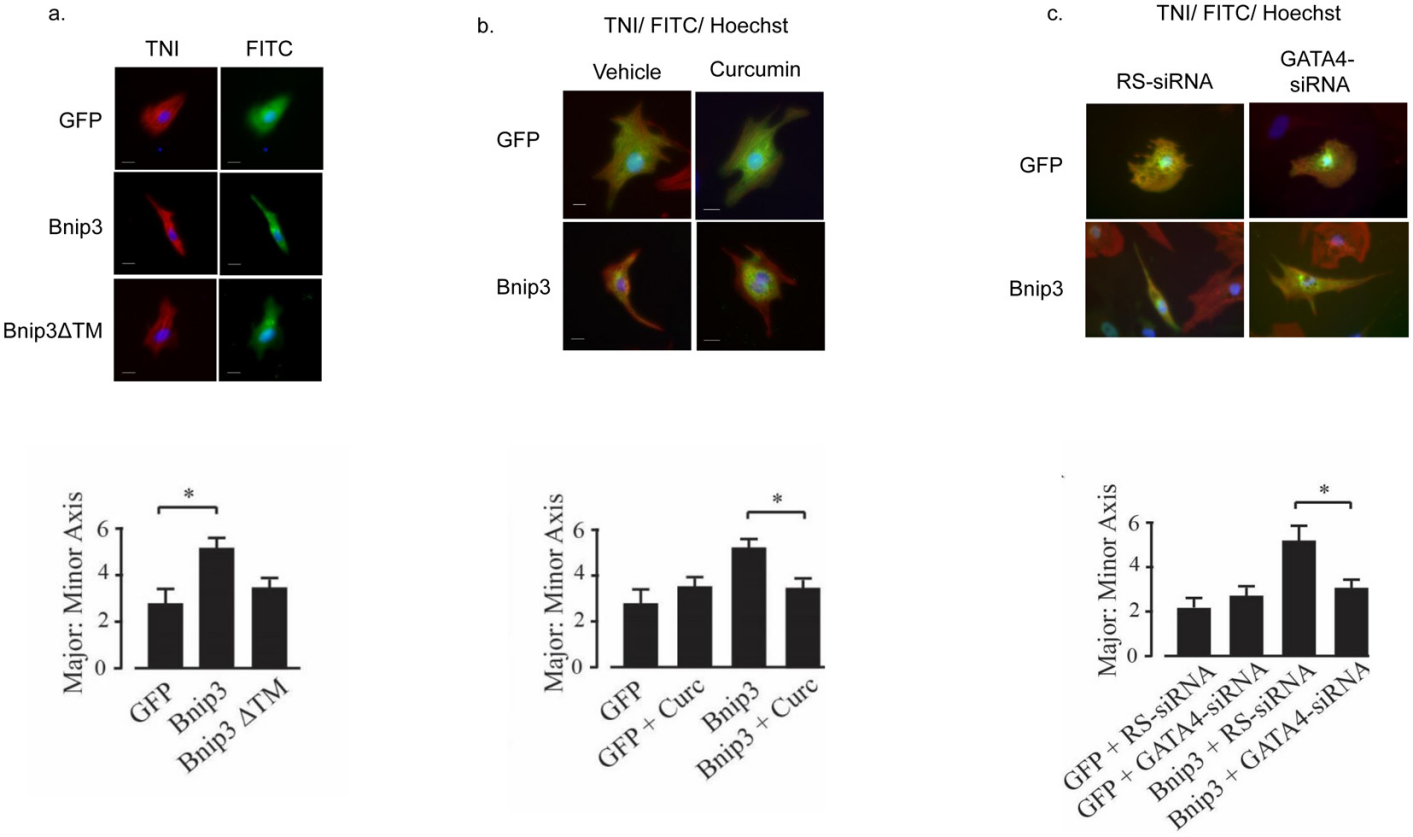
Curcumin and GATA4 sensitive changes in myocyte morphology. In (A) cardiac myocytes were transfected with expression plasmids for GFP, Bnip3, or Bnip3ΔTM and the ratio of the cell length to width determined. Myocyte identity was confirmed by troponin (TNI) immunocytochemistry. Quantitative analysis of the major to minor axis ratio is shown below. The effect of curcumin (Curc) on the major to minor axis ratio is shown in (B) with quantitative analysis of the major to minor axis ratio again shown below. The effect of GATA4 and random (RS) siRNA on the major to minor axis ratio is shown in (C) with quantitative analysis of the major to minor axis ratio also shown below. Results are mean ± S.E.M., significantly different then controls (p < 0.05), n = 3.

Previous studies have described p300-regulation of GATA4 and MEF2 transcription factors and their roles in cardiac myocyte morphology, cardiac development and hypertrophy [18]. Therefore we hypothesized that Bnip3 overexpression may alter cardiac myocyte morphology through its regulation of p300 and GATA4. To test this cardiac myocytes were transfected with vectors expressing GFP, Bnip3 or a Bnip3 transmembrane deletion mutant (ΔTM) and the morphology of transfected myocytes quantified by morphometry. As shown in Fig 6A, Bnip3 overexpression was associated with a 2-fold increase in cell length (2.1 ± 0.2 increase in major: minor axis; p < 0.05) without hypertrophy. Morphology was not significantly affected by Bnip3-ΔTM transfection. To determine whether the change in morphology required p300 and GATA4, Bnip3 transfected myocytes were treated with curcumin to inhibit p300, or with a GATA4 specific siRNA and the measurements repeated. As shown in Fig 6B and 6C, the morphological changes conferred by Bnip3 overexpression were significantly blocked by curcumin treatment or knockdown of GATA4. The results suggest that Bnip3 confers myocyte elongation by mechanism(s) that involve p300 and GATA4.

### Bnip3 overexpression in the heart induces dilated cardiomyopathy

**Fig 7:**
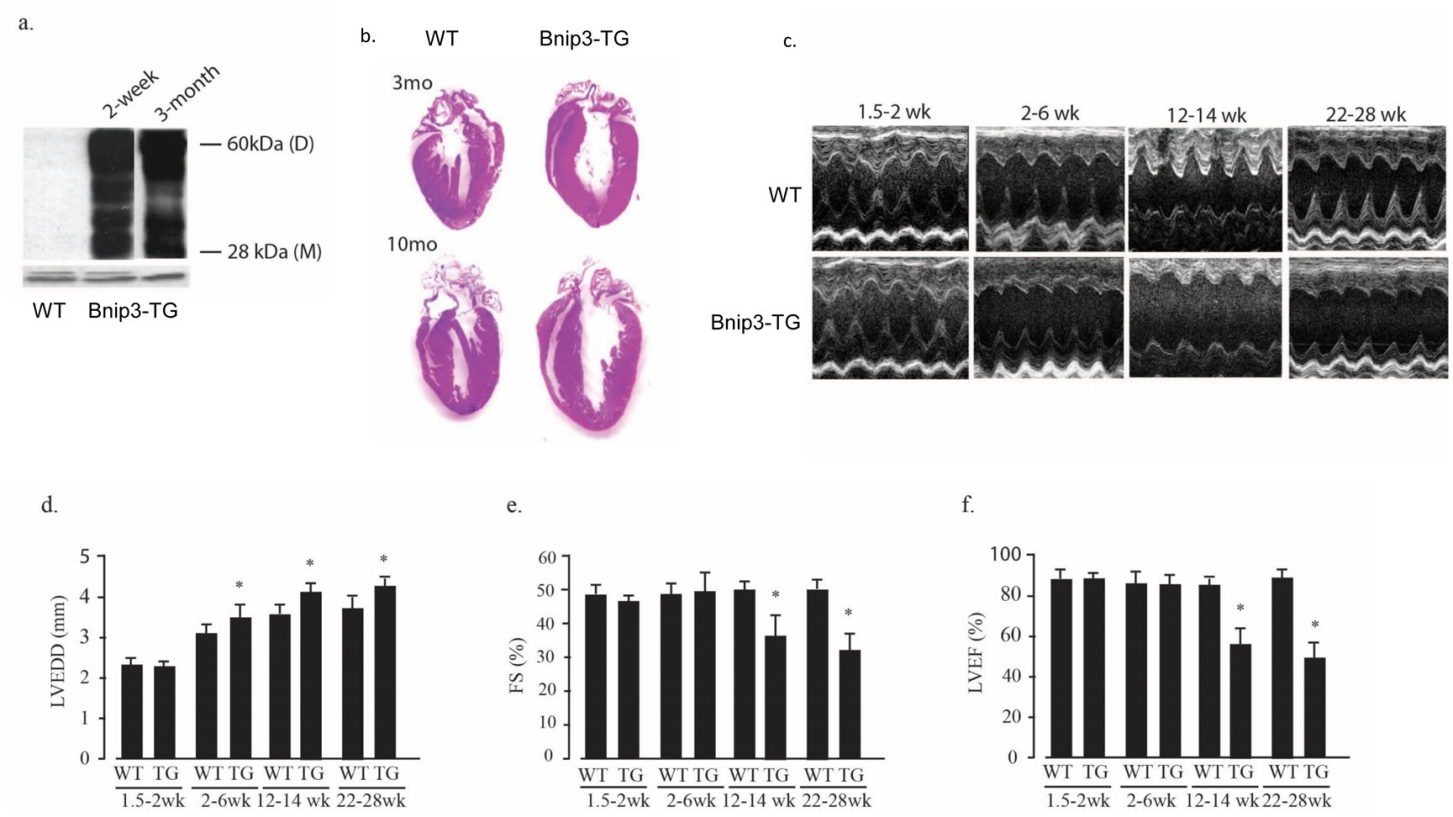
Bnip3 dependent ventricular dilation and contractile dysfunction. (A) Bnip3 protein levels were determined in heart lysates from wild type (WT) and α-MHC-Bnip3 transgenic mice (Bnip3-TG) at the indicated ages. (B) Hematoxylin and eosin—stained coronal heart sections from 3 and 10 month old wild type (WT) and Bnip3-TG mice. Examples of short axis M-mode echocardiograms of WT and Bnip3-TG mice at the indicated ages are shown in (C). (D-F) echocardiographically determined left ventricular end diastolic diameter (LVEDD), left ventricular fractional shortening (FS) and left ventricular ejection fraction (LVEF) at the indicated ages. Results are mean ± S.E.M. (* p < 0.05); n = 5.

The results of Figs 1–6 show that Bnip3 activation by HA in isolated cardiac myocytes increased p300-acetyltransferase activity and acetylation of histones and GATA4. In previous work we reported that p300 promotes hypertrophy and dilated cardiomyopathy in transgenic mice [18]. This was associated with enhanced activity and acetylation of GATA4 and MEF2 and a small increase of basal histone H3 acetylation. To determine whether Bnip3 drives similar p300-GATA4-dependent events in vivo, we generated transgenic mice that over-express heart-specific Bnip3 directed by the α-MHC promoter. In agreement with a previous report [15], we found that hearts from Bnip3 overexpressing mice displayed early-onset ventricular dilation followed by contractile abnormalities (Fig 7). Bnip3 transgenic hearts expressed > 50-fold higher levels of Bnip3 compared with wild type hearts as early as 2 weeks of age, and sustained this level of Bnip3 expression as adults (Fig 7A). The Bnip3-TG phenotype included progressive enlargement of the heart and chamber dilation without overt hypertrophy (Fig 7B). These results were supported by echocardiography that indicated progressive chamber dilation that was evident as early as 2–6 weeks of age and was followed by diminished systolic performance with significant reductions in fractional shortening and ejection fraction at 3 months of age (Fig 7C–7F).

### Ventricular dilation proceeds programmed cell death in Bnip3-TG hearts

**Fig 8:**
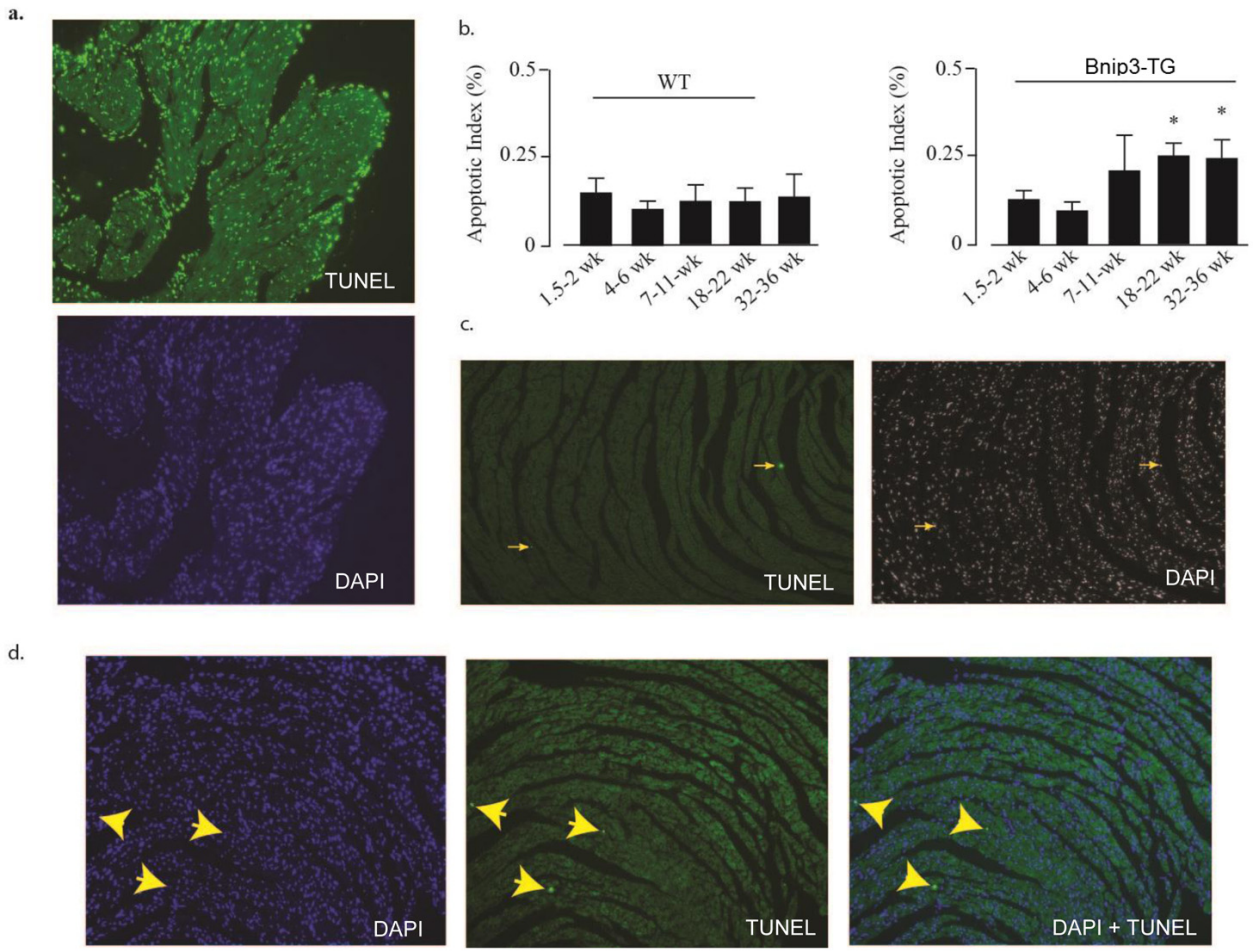
Apoptosis in aged but not young Bnip3-TG mice. TUNEL staining was implemented as described in Methods. (A) The sensitivity of the TUNEL assay was demonstrated by treating heart sections with DNAase I to induce TUNEL positive DNA strain breaks; top panel TUNEL, bottom panel DAPI; >80% of nuclei are TUNEL-positive (data not shown). (B) Apoptotic indices of wild type (WT) and Bnip3-TG mice were determined by TUNEL at the indicated times as described in Methods. Examples of TUNEL staining in heart sections from 5-week old wild type (C) and Bnip3-TG mice (D). Nuclei were co-stained with DAPI. Yellow arrows point to TUNEL positive nuclei and the corresponding DAPI stained nuclei. Results in (B) are mean ± S.E.M.; * p < 0.05, comparing apoptotic indices with 4–6 weeks (n = 5).

It is widely acknowledged that programmed cell death parallels and contributes to the progression of heart failure [29, 30]. Therefore we were interested in determining whether apoptotic indices were elevated in Bnip3-TG hearts and if so whether they correlated temporally with ventricular dilation and systolic dysfunction. Consistent with a previous report [15] we found elevated apoptotic indices in Bnip3-TG hearts (Fig 8). However, apoptotic indices of Bnip3-TG hearts did not differ from wild type hearts when mice were less than 7 weeks old (Fig 8B) despite full activation of the Bnip3 transgene at 2 weeks after birth (see Fig 7A). The increase in apoptotic indices over time was associated with the development of systolic dysfunction. Therefore programmed cell death as reflected by TUNEL analysis in this model is age-dependent and not apparent in young mice despite high Bnip3 overexpression.

### Enhanced GATA4 acetylation and p300 interaction in Bnip3-TG hearts

**Fig 9:**
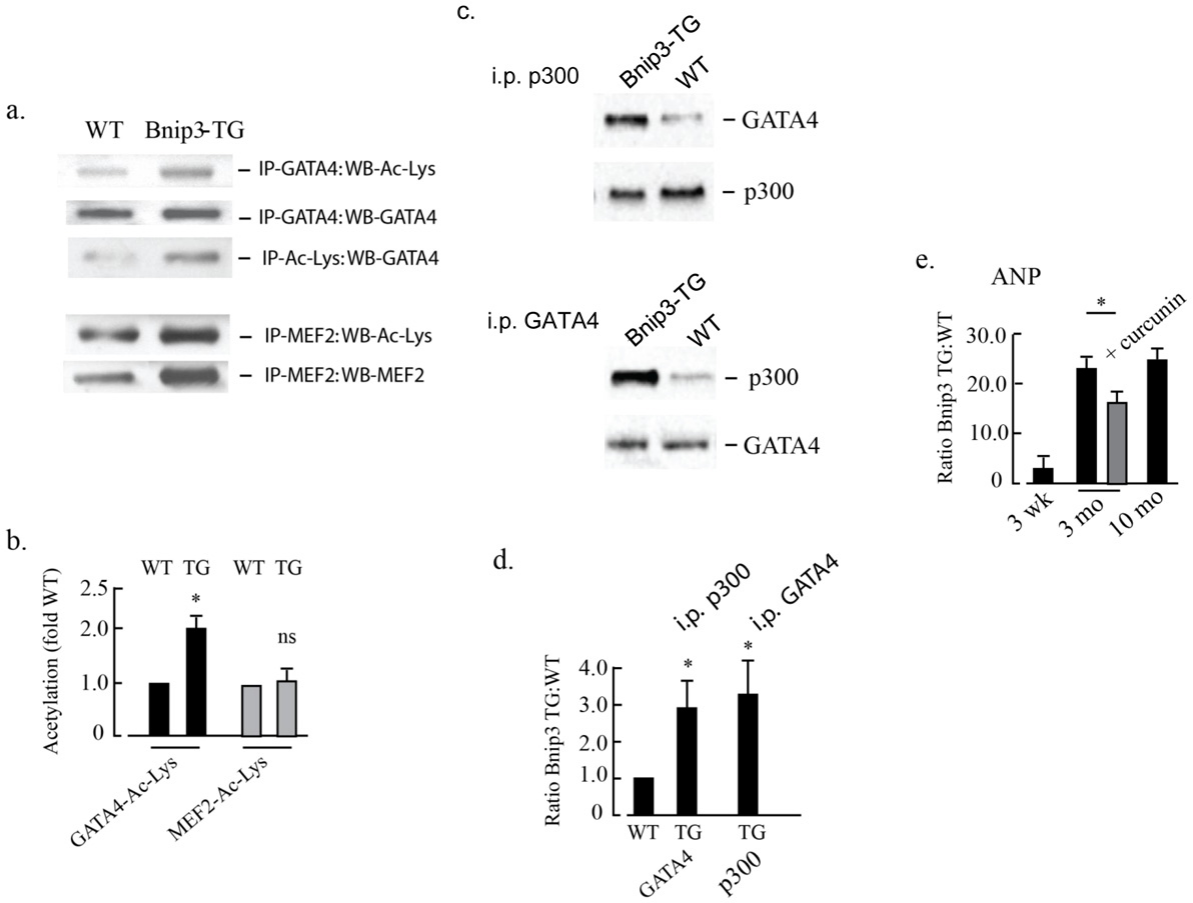
Bnip3 associates with p300 and GATA4 leading to altered gene expression. In (A) GATA4, MEF2 and acetylated lysine were immunoprecipitated from total heart lysates generated from wild type (WT) and Bnip3-TG mice. Co-immunoprecipitated (IP) proteins were determined by western blot (WB) analysis as indicated. Quantitation of the changes in GATA4 and MEF-2 acetylation levels are shown in (B). In (C) nuclear extracts were made from heart lysates and reciprocal immunoprecipitation of p300 and GATA4 performed as indicated. Quantitation of p300 and GATA4 reciprocal immunoprecipitation is shown in (D). In (E) transcript levels of atrial natriuretic protein (ANP) in Bnip3-TG hearts ± curcumin relative to wild type hearts was determined by RT-PCR at the indicated ages. All results are mean ± S.E.M., (p < 0.05), n = 3.

Reciprocal co-immunoprecipitation reactions were performed on total myocardial extracts from wild type or Bnip3-TG mice to quantify acetylation of GATA4 and MEF2. As shown in Fig 9A and 9B GATA4 acetylation was significantly increased when Bnip3-TG extracts were used for pull-down relative to wild type hearts using either GATA4 or acetyl-lysine antibodies (panels 1–3). In contrast, MEF2 appeared to be acetylated similarly in both wild type and Bnip3-TG hearts. MEF2 protein as well as acetylated lysine increased in parallel in Bnip3-TG hearts but the ratio of MEF2:acetylated lysine was unchanged (Fig 9A, panels 4 and 5). Results of reciprocal co-immunoprecipitations of GATA4 and p300 from nuclear lysates are shown in Fig 9C and 9D. P300 and GATA4 interactions were increased >3-fold in nuclear lysates from Bnip3-TG hearts compared with wild type hearts. Together these results suggest enhanced interaction between p300 and GATA4 in Bnip3-TG hearts, similar to our findings with isolated cardiac myocytes.

To further characterize functional interactions between Bnip3-p300 and GATA4, we measured the transcript level of atrial natriuretic protein (ANP), a gene that is regulated by p300-GATA4 and a marker of heart failure [31–33]. As shown in Fig 9E, ANP transcripts were increased within 3 month of age and remained elevated at 10 months in the Bnip3-TG hearts. ANP expression was significantly reduced by curcumin treatment.

### Curcumin reduces cardiac dysfunction in Bnip3-TG mice

**Fig 10:**
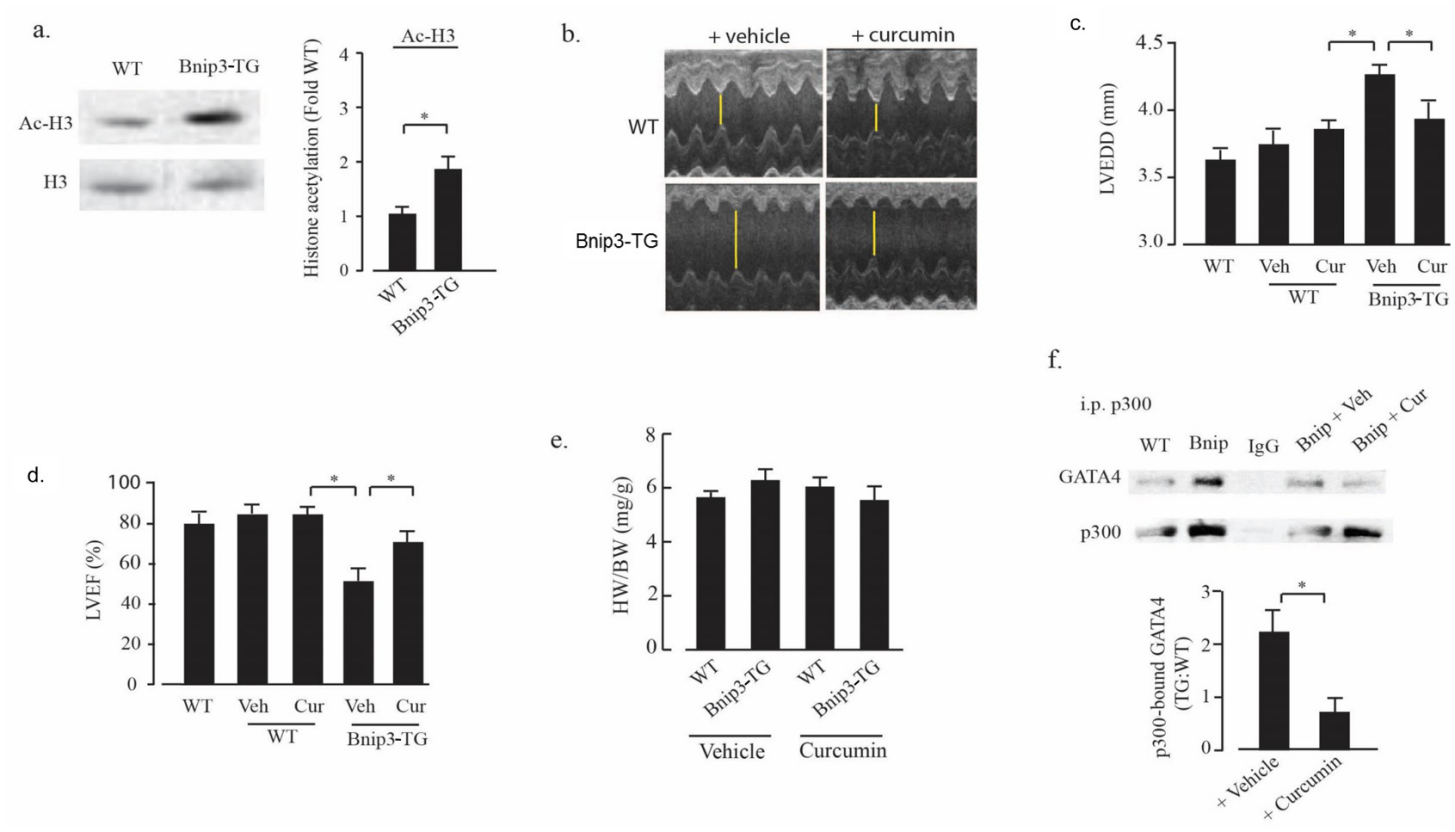
Curcumin treatment improves Bnip3-TG cardiac performance. (A) Histone H3 acetylation (Ac-H3) levels in left ventricular lysates from wild type (WT) and Bnip3-TG mice were measured and quantified as described in Methods. (n = 3; * p < 0.05). Example images of short axis M-mode echo cardiograms of WT and Bnip3-TG mice treated with either vehicle or curcumin as described in Methods (B). The effect of vehicle (Veh) or curcumin (Cur) treatment on echocardiographically determined left ventricular ejection fraction (LVEF), left ventricular end diastolic diameter (LVEDD), and heart weight to body weight (HW/BW) ratio of WT and Bnip3-TG mice is shown in (C–E). In (F) p300 was immunoprecipitated from the hearts of wild type (WT), Bnip3-TG, and Bnip3-TG mice treated with vehicle (Veh) or curcumin (Cur) and the association of p300 with GATA4 determined by western blot analysis. Quantitation of p300 bound GATA4 is shown below (*p < 0.05, n = 5). Results are mean ± S.E.M.

Based on the effects of Bnip3 overexpression on p300 and myocyte morphology in vitro, and the increased levels of p300 and acetylated GATA4 in Bnip3-TG mice (Fig 9), we hypothesized that enhanced activity of p300/GATA4 in Bnip3-TG mice contributes to the progressive cardiomyopathy described in Fig 7. Consistent with this, we found that acetylated histone H3 levels were significantly higher in extracts from Bnip3-TG hearts when compared to wild type littermates (Fig 10A). Furthermore, we found that inhibition of p300-acetyltransferase with curcumin significantly reduced left ventricular dilation and deterioration of ejection fraction of Bnip3-TG hearts but had no effect on contractile parameters of wild type mice (Fig 10B–10D). Curcumin treatment did not affect heart or body weight of wild type or Bnip3-TG mice (Fig 10E) and the protective effects of curcumin were paralleled by reductions of co-immunoprecipitated GATA4 and p300 in extracts from Bnip3-TG hearts (Fig 10F). These results implicate curcumin-sensitive p300-GATA4 in the progression of contractile dysfunction and DCM in Bnip3-TG hearts.

### Binp3 binds the TORC1 regulator Rheb

**Fig 11:**
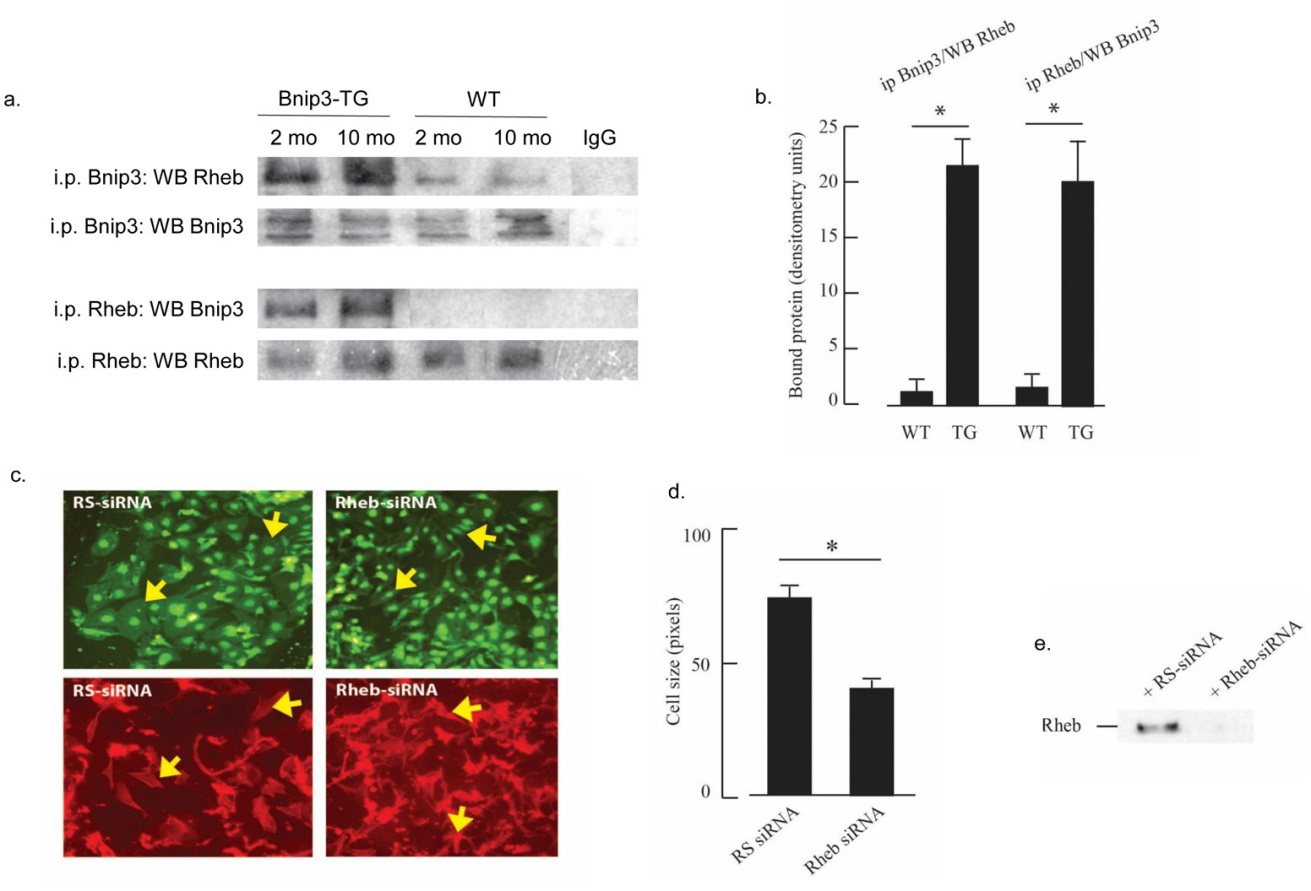
Rheb prevents cardiac hypertrophy and interacts with Bnip3. Myocardial lysates from Bnip3-TG and wild type (WT) mice were prepared and processed for reciprocal Bnip3 and Rheb immunoprecipitation as described in Methods. Representative western blots showing immunoprecipitation (i.p.) and western blot (WB) antibodies are shown in (A). Quantification of western blot immunoprecipitation is shown in (B) (n = 3; * p < 0.002). (C) Representative plates of neonatal rat cardiac myocytes after transfection with random sequence (RS) or Rheb siRNA and exposure to serum as a hypertrophy stimulus as described in Methods. Top panels GFP; bottom phalloidin (red); arrows indicate hypertrophied myocytes in RS panels and reduced myoplasm area with Rheb-siRNA treatment. (D) Quantification of cell area from ~100 myocytes per group as described in Methods (n = 3; * p < 0.002). (E) Representative western blot shown efficient KD of Rheb by siRNA. All results are mean ± S.E.M.

Our results as well as others have shown that p300 and GATA4 promote hypertrophic myocardial growth [18], therefore the absence of hypertrophic growth concomitant with activated p300 and GATA4 in isolated myocytes or Bnip3-TG hearts presents a paradox. Hypertrophic growth is associated with increased activity of the Akt-TORC1 pathway and it was recently shown that genetic ablation of Rheb, an immediate upstream regulator of TORC1, blocks such growth [34, 35]. Another report demonstrated that Bnip3 bound and sequestered Rheb in HEK 293 cells [6]; therefore we hypothesized that Bnip3 sequestration of Rheb may prevent ventricular hypertrophy in Bnip3-TG mice. To test this hypothesis we used reciprocal co-immunoprecipitation reactions to quantify the interaction between Rheb and Bnip3 in hearts from wild type and Bnip3-TG mice. As shown in Fig 11A and 11B, co-immunoprecipitation reactions of Bnip3 and Rheb confirmed an interaction between Bnip3 and Rheb in myocardial tissue. To investigate a role for Rheb in cardiac myocyte hypertrophy we used siRNA to selectively knockdown Rheb in isolated myocytes and then exposed cultures to serum to induce hypertrophy. As shown in Fig 11C and 11D), Rheb knockdown significantly reduced myocyte size confirming the role for Rheb in hypertrophic growth. Therefore it seems possible that Bnip3-sequestration of Rheb may be responsible for preventing hypertrophy in Bnip3-TG mice despite enhanced p300, GATA4 and histone acetylation.

## Discussion

Here we describe a novel activity of Bnip3 that is independent of mitochondrial function and programmed cell death, and involves binding and activation of p300-acetyltransferase with downstream effects on transcription and histone acetylation. In isolated cardiac myocytes histone acetylation was significantly increased in a Bnip3-dependent manner when myocytes were exposed to hypoxia with acidosis. Inhibition of p300 with curcumin but not blockade of programmed cell death prevented enhanced histone acetylation. P300-specific acetyltransferase activity and total HAT activity were elevated in a Bnip3-dependent manner during hypoxia-acidosis suggesting a regulatory role for Bnip3 in acetyltransferase activation. Furthermore recombinant Bnip3 protein was sufficient to increase HAT activity in nuclear lysates isolated from normoxic cardiac myocytes, suggesting a direct effect of Bnip3 on HAT activity independent of hypoxia/acidosis or mitochondrial Bnip3. Co-immunoprecipitation reactions revealed enhanced p300-dependent acetylation of the cardiac-specific transcription factor GATA4 in extracts from myocytes that overexpress Bnip3. Transfection of cardiac myocytes with full-length Bnip3 cDNA conferred changes in myocyte shape that were prevented by curcumin or by siRNA-knockdown of GATA4. Bnip3 regulated morphological changes did not involve hypertrophy of isolated myocytes, a result that differs from our previous finding that p300 overexpression induces cardiac myocyte hypertrophy [18]. These seemingly contradictory findings suggest that the effects of Bnip3 on myocyte morphology involves more than simply enhanced p300 activation [18] and may reflect additional actions of Bnip3 in parallel. Bnip3 interactions with ER, Rheb and specific promotors have been reported previously in glioblastoma cells [6,7,16]

Our studies using Bnip3 overexpressing transgenic mice confirm a previous report that cardiac-specific overexpression of Bnip3 results in progressive contractile dysfunction and dilated cardiomyopathy (DCM) [26]. DCM was accompanied by enlargement of the myocardium and chamber dilation but without apparent wall thinning or fibrosis that occurs when DCM is driven primarily by program cell death [34, 36].

Results described in Figs 7, 9 and 10 show that the effects of Bnip3 overexpression on p300/GATA4 seen in isolated cardiac myocytes are also apparent in intact Bnip3-TG hearts. In co-immunoprecipitation assays from nuclear fractions, p300 pulled down 3-times more GATA4 when the extracts were from Bnip3-TG compared with wild type hearts, and the same results were seen in reciprocal pull down of p300 by GATA4 (Fig 9C and 9D). Therefore elevated p300 activity in Bnip3-TG hearts may contribute to the curcumin-sensitive contractile dysfunction and DCM (Fig 10). Bnip3-TG hearts expressed high levels of ANP (Fig 9), a p300/GATA4-regulated gene and a marker of DCM and heart failure [37–40]. Notably both contractile dysfunction and ANP expression in Bnip3-TG mice were both sensitive to curcumin treatment supporting a role for p300 in this phenotype. Such sensitivity would not be predicted for a pathway of cardiomyopathy driven solely by program cell death.

Two main lines of evidence support roles for enhanced p300 activity in the initiation and progression of DCM in Bnip3-TG mice. (1) Contractile dysfunction was alleviated by treatment with curcumin in parallel with reduced binding of p300 and GATA4, and lower ANP expression. (2) Increased programmed cell death in Bnip3-TG hearts was evident only after 7-weeks of age and coincided with, rather than preceded contractile dysfunction and DCM. In our hands apoptotic indices in heart sections of Bnip3-TG mice were elevated to > 0.25% (2-3-fold higher than corresponding wild type mice) in mice at 7-weeks or older. Our apoptotic indices numbers are within the range described by Wencker et al [30] and Zhang et al [2] for mice overexpressing caspase or mitochondrial mutations respectively but lower than those reported by Diwan et al [15]. The absence of elevated program cell death in young (i.e. 4–6 weeks) Bnip3-TG mice suggests that the death function of Bnip3 is not active despite full activation of the transgene as early as 2-weeks of age. Therefore we propose that specific transcriptional changes associated with Bnip3, p300 and GATA4 contribute to early ventricular dilation that is followed by contractile dysfunction in Bnip3-TG mice. Furthermore myocyte death in Bnip3 transgenic hearts may be exacerbated by stress imposed on the myocytes caused by contractile dysfunction, with program cell death contributing increasingly to the DCM phenotype as the mice age. The possibility of a Bnip3 activation step is supported by the studies of Diwan et al [15] and Chaanine et al [16] wherein program cell death is activated during acute myocardial infarction and pressure overload heart failure in mice that already express elevated Bnip3.

The phenotype of Bnip3-TG overexpression described here more closely resembles that conferred by the overexpression of MEF2 transcription factors [37, 41]. Whereas MEF2 and GATA4 genes are each induced by hypertrophic stimuli, most studies associate GATA4 with developmental and adaptive hypertrophic growth, while MEF2A/C factors are more closely linked with pathologic hypertrophy and DCM [32, 41]. In our studies myocyte shape changes and DCM associated with Bnip3 overexpression correlated with elevated activities of p300 and GATA4 but less so with MEF2. Multiple factors in addition to MEF2 and GATA4 contribute to hypertrophy and DCM; these include serum response factor, AP-1, nuclear factor of activated T cells, NFkB, SMAD transcription factors, Nkx2-5, myocardin and Akt-TORC1, (reviewed in [42, 43]). Bnip3 may also exert selective regulation of these factors through p300 or directly as evidenced by its effect on TORC1/Rheb. A unifying feature of our observations was that curcumin inhibited the molecular and physiological effects of Bnip3 overexpression in vitro and in vivo thus supporting a central role for p300. However we do not know the precise targets of p300 beyond GATA4 in this context.

## Conclusion

In conclusion, we have shown that Bnip3 activates p300 and associated acetylation reactions to levels that were shown previously to cause maladaptive hypertrophy and dilated cardiomyopathy [18, 24, 25]. We propose that the death-inducing functions of Bnip3 are inactive in the hearts of young (< 6 weeks) Bnip3-TG mice despite high expression of the transgene. Rather the death program is activated at later times, perhaps by the accumulation of metabolic and other stress factors that are associated with progressive contractile dysfunction. It is well known that p300-dependent pathways contribute to cellular reprogramming subsequent to myocardial stress including post-acute myocardial infarction remodeling and hypertrophy [15,16,18,26,28,32,44]. Based upon the data presented such pathways are predicted to be active whenever Bnip3 is induced by stressors such as physical, metabolic or vascular disturbance, conditions which if not corrected may also lead to a secondary activation step(s) for Bnip3 programmed death [1, 8, 10, 44, 45].
